# Long COVID in a prospective cohort of home-isolated patients

**DOI:** 10.1038/s41591-021-01433-3

**Published:** 2021-06-23

**Authors:** Bjørn Blomberg, Kristin Greve-Isdahl Mohn, Karl Albert Brokstad, Fan Zhou, Dagrun Waag Linchausen, Bent-Are Hansen, Sarah Lartey, Therese Bredholt Onyango, Kanika Kuwelker, Marianne Sævik, Hauke Bartsch, Camilla Tøndel, Bård Reiakvam Kittang, Bergen COVID-19 Research Group, Anders Madsen, Geir Bredholt, Juha Vahokoski, Elisabeth Berg Fjelltveit, Amit Bansal, Mai Chi Trieu, Sonja Ljostveit, Jan Stefan Olofsson, Nina Ertesvåg, Helene Heitmann Sandnes, Anette Corydon, Hanne Søyland, Marianne Eidsheim, Kjerstin Jakobsen, Nina Guldseth, Synnøve Hauge, Rebecca Jane Cox, Nina Langeland

**Affiliations:** 1 Department of Clinical Science, University of Bergen, Bergen, Norway; 2 National Advisory Unit for Tropical Infectious Diseases, Haukeland University Hospital, Bergen, Norway; 3 Department of Medicine, Haukeland University Hospital, Bergen, Norway; 4 Influenza Centre, Department of Clinical Science, University of Bergen, Bergen, Norway; 5 Broegelmann Research Laboratory, Department of Clinical Science, University of Bergen, Bergen, Norway; 6 Department of Safety, Chemistry and Biomedical Laboratory Sciences, Western Norway University of Applied Sciences, Bergen, Norway; 7 Bergen Municipality Emergency Clinic, Bergen, Norway; 8 Department of Medicine, Haraldsplass Deaconess Hospital, Bergen, Norway; 9 Mohn Medical Imaging and Visualization Centre, Department of Radiology, Haukeland University Hospital, Bergen, Norway; 10 Department of Informatics, University of Bergen, Bergen, Norway; 11 Department of Paediatrics, Haukeland University Hospital, Bergen, Norway; 12 Department of Research and Innovation, Haukeland University Hospital, Bergen, Norway; 13 Department of Microbiology, Haukeland University Hospital, Bergen, Norway

**Keywords:** Viral infection, Epidemiology, Fatigue

## Abstract

Long-term complications after coronavirus disease 2019 (COVID-19) are common in hospitalized patients, but the spectrum of symptoms in milder cases needs further investigation. We conducted a long-term follow-up in a prospective cohort study of 312 patients—247 home-isolated and 65 hospitalized—comprising 82% of total cases in Bergen during the first pandemic wave in Norway. At 6 months, 61% (189/312) of all patients had persistent symptoms, which were independently associated with severity of initial illness, increased convalescent antibody titers and pre-existing chronic lung disease. We found that 52% (32/61) of home-isolated young adults, aged 16–30 years, had symptoms at 6 months, including loss of taste and/or smell (28%, 17/61), fatigue (21%, 13/61), dyspnea (13%, 8/61), impaired concentration (13%, 8/61) and memory problems (11%, 7/61). Our findings that young, home-isolated adults with mild COVID-19 are at risk of long-lasting dyspnea and cognitive symptoms highlight the importance of infection control measures, such as vaccination.

## Abstract (web summary)

Analysis of a prospectively enrolled cohort of patients with SARS-CoV-2 infections in Bergen, Norway, reveals a high proportion of patients who experienced long COVID symptoms at 6 months, despite being relatively young and having only mild to moderate acute COVID-19 symptoms.

## Main

The respiratory tract is the site of severe acute respiratory syndrome coronavirus 2 (SARS-CoV-2) entry and infection; however, COVID-19 is a complex systemic disease, affecting the cardiovascular, renal, hematologic, gastrointestinal and central nervous systems^1^. As evidence emerges of predominantly lasting impairment of lung function related to fibrosis, more data on the long-term effects of COVID-19 on other organs are required^2^. A plethora of symptoms persist in patients surviving severe COVID-19 (refs. ^3,4^), and a long COVID syndrome has been proposed^5,6^. However, the severity and duration of symptoms remain largely unknown. Chronic fatigue occurred after SARS infection in 2003 (ref. ^7^), and it is well known in the aftermath of a spectrum of infectious diseases^8–13^. Before the SARS-CoV-2 pandemic, patient management in intensive care was frequently associated with mental and physical decline, and this could partially explain long COVID in patients with severe illness^14^. However, the burden of long COVID in mild to moderately ill patients is not well defined. We assessed persistent symptoms 6 months after initial COVID-19 in a prospective cohort of hospitalized and home-isolated patients from the first pandemic wave in Bergen, Norway.

## Results

**Fig. 1:**
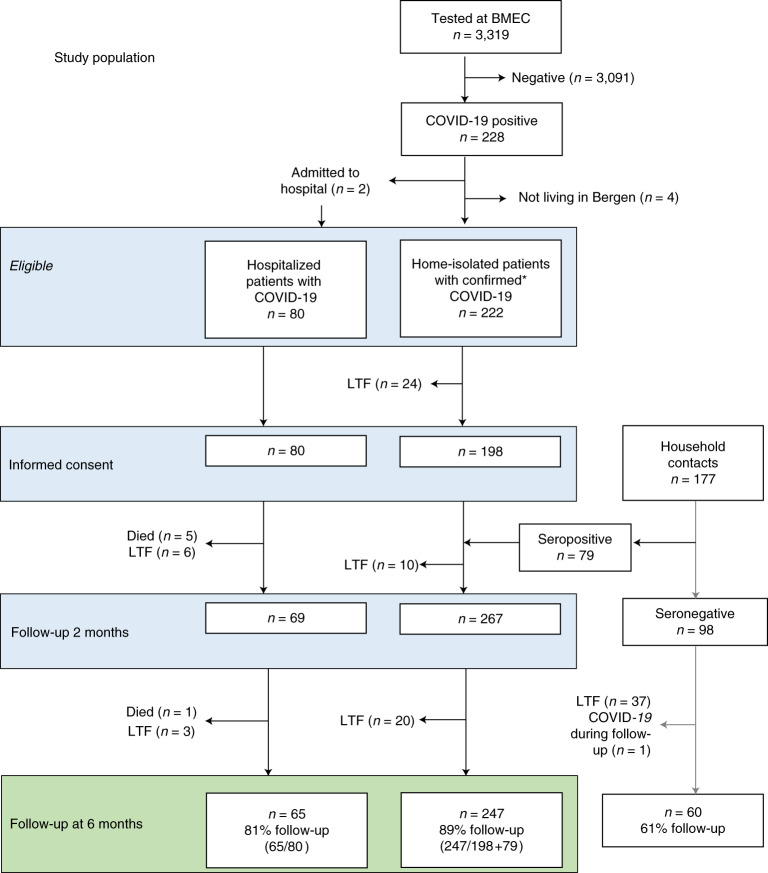
Study population flow chart. Number of participants eligible for inclusion at baseline, blood sampling at 2 months and assessment and fatigue evaluation at 6 months. BMEC, Bergen Municipality Emergency Clinic; LTF, lost to follow-up.

All patients diagnosed at the only centralized testing facility in the city of Bergen were invited to participate, as well as all patients admitted to the city’s two hospitals: Haukeland University Hospital and Haraldsplass Deaconess Hospital. Recruitment commenced with the first diagnosed home-isolated and the first hospitalized patients; 92% of patients during the first pandemic wave agreed to participate. The objective was to identify factors and biomarkers associated with long-term complications. From 28 February to 4 April 2020, we consecutively recruited 357 patients who were positive for SARS-CoV-2. We collected demographic and clinical data as well as blood samples. Household members of patients who tested positive were included to ensure completeness of the cohort, and their infection was diagnosed by SARS-CoV-2-specific antibodies at 2 months^15^. At 6-month follow-up, the study population available for analysis comprised 312 patients, of whom 247 were home-isolated and 65 were hospitalized (Fig. 1).

The median age of the study population was 46 years (interquartile range (IQR) 30–58 years) with 51% (160/312) women. Forty-four percent had comorbidities (137/312), the most frequent being chronic lung disease (12%, 38/312, 34 with asthma), hypertension (11%, 35/312), chronic heart disease (7%, 22/312), rheumatic diseases (6%, 20/312), diabetes (4%, 13/312) and immunosuppressive conditions (4%, 11/312). In 272 patients who recorded symptoms during acute disease, fatigue (90%, 244/272), cough (71%), headache (64%), myalgia (58%) and dyspnea (55%) were the most frequent, whereas only 21% had fever. Hospitalized patients were older than home-isolated patients, had higher body mass index (BMI) and had more comorbidities, including chronic lung disease, chronic heart disease, hypertension and diabetes (Table 1).

**Table 1:** Characteristics of study population available for follow-up at 6 months

	Seronegative exposed controls	All patients with COVID-19	Hospitalized COVID-19	Home-isolated COVID-19
	% (*n*)	% (*n*)	% (*n*)	% (*n*)
	*N* = 60	*N* = 312	*N* = 65	*N* = 247
Female sex	63% (38)	51% (160)	46% (30)	53% (130)
Age, median (IQR)	29 (14–48)	46 (30–58)	55 (45–68)	43 (27–55)
Age categories				
0–15 years	28% (17)	5% (16)	0% (0)	6% (16)
16–30 years	23% (14)	21% (65)	6% (4)	25% (61)
31–45 years	22% (13)	22% (69)	17% (11)	23% (58)
46–60 years	25% (15)	29% (90)	35% (23)	27% (67)
>60 years	2% (1)	23% (72)	42% (27)	18% (45)
BMI, median (IQR)	23.3 (20.9–25.6)	24.6 (22.8–27.3)	27.0 (24.1–29.9)	24.3 (22.5–26.5)
Any comorbidity^a^	15% (9)	44% (137)	69% (45)	37% (92)
Asthma, COPD^b^	2% (1)	12% (38)	22% (14)	10% (24)
Hypertension	0% (0)	11% (35)	25% (16)	8% (19)
Chronic heart disease	0% (0)	7% (22)	18% (12)	4% (10)
Rheumatic disease	2% (1)	6% (20)	12% (8)	5% (12)
Diabetes mellitus	3% (2)	4% (13)	9% (6)	3% (7)
Immunosuppression	0% (0)	4% (11)	8% (5)	2% (6)
Current or prior smoker^c^	19% (11/57)	31% (96/310)	39% (25/64)	29% (71/246)
Severity of disease^d^				
Asymptomatic (1)	53% (30/57)^e^	2% (5/312)	-	2% (5/247)
Home-isolated with symptoms (2)	47% (27/57)^e^	78% (242/312)	-	98% (242/247)
Hospitalized without medical needs (3)		-	-	-
Hospitalized with medical needs (4)		10% (31/312)	48% (31/65)	-
Hospitalized needing O_2_ (5)		8% (24/312)	37% (24/65)	-
Hospitalized needing NIV (6)		1% (4/312)	6% (4/65)	-
Hospitalized needing respirator (7)		2% (6/312)	9% (6/65)	-
Dead (8)		-	-	-
Severity of illness, median (IQR)	-	2 (2–2)	5 (4–5)	2 (2–2)
Days in hospital, median (IQR)	-	0 (0–0)	6 (2–8)	0 (0–0)
Spike antibodies^f^	Negative^g^	3.9 (3.8–4.0)	4.6 (4.4–4.8)	3.7 (3.6–3.8)
Microneutralizing antibodies^f^	-	2.0 (2.0–2.1)	2.9 (2.7–3.1)	1.8 (1.8–1.9)

COPD, chronic obstructive pulmonary disease; O_2_, supplemental oxygen; NIV, non-invasive ventilation.

^a^Comorbidities are listed in descending order of frequency. Participants were asked if they had any comorbidities; if yes, they were asked about the following specific comorbidities: asthma, chronic obstructive lung disease, chronic heart disease, hypertension, chronic liver disease, kidney disease, neuromuscular disease, dementia, rheumatic disease, active cancer, other severe chronic disease and immunosuppressive conditions, including genetic immunodeficiency, HIV, organ transplant and cytostatic or other immunosuppressive treatment.

^b^34 of 38 had asthma.

^c^Not known for five patients.

^d^Eight-step severity score modified after Beigel et al. (ref. ^17^).

^e^Missing data for three patients.

^f^Measured 2 months after initial illness; log_10_ titers of IgG antibodies, means and 95% confidence intervals.

^g^Below the assay detection limit; only positive samples were run in the microneutralization assay.

Sixty-one percent (189/312) of the total patient population had persistent symptoms 6 months after initial COVID-19 illness, with the most common symptoms being fatigue (37%), difficulty concentrating (26%), disturbed smell and/or taste (25%), memory problems (24%) and dyspnea (21%). Whereas the frequency of most symptoms increased with age in the study population, disturbed smell and/or taste was more frequent in people younger than 46 years old (Table 2). Thirty-nine percent of the study population, commonly children and young adults, had no symptoms at 6 months. Even among the 247 home-isolated patients, 55% (136/247) experienced persistent symptoms at 6 months, most commonly fatigue (30%), disturbed taste and/or smell (27%), concentration impairment (19%), memory loss (18%) and dyspnea (15%) (Table 2).

**Table 2:** Long-term complications by age group in 247 home-isolated patients with COVID-19 at 6-month follow-up

Characteristic	All	0–15 years	16–30 years	31–45 years	46–60 years	Over 60 years
	% (*n*/*N*)	% (*n*)	% (*n*)	% (*n*)	% (*n*)	% (*n*)
	*N* = 247	*N* = 16	*N* = 61	*N* = 58	*N* = 67	*N* = 45
Age, median (IQR)	43 (27–55)	8 (6–12)	24(22–27)	37 (34–41)	53 (49–55)	67 (63–73)
Female gender	53% (131/247)	56% (9)	54% (33)	52% (30)	52% (35)	53% (24)
Status at 6 months						
Any symptoms	55% (136/247)	13% (2)*	52% (32)	59% (34)	61% (41)	60% (27)
Fever	2% (4/247)	0% (0)	0% (0)	5% (3)	1% (1)	0% (0)
Cough	6% (15/247)	0% (0)	0% (0)	9% (5)	4% (3)	16% (7)
Dyspnea	15% (38/247)	0% (0)	13% (8)	17% (10)	18% (12)	18% (8)
Palpitations	6% (15/247)	0% (0)	3% (2)	7% (4)	9% (6)	7% (3)
Stomach upset	6% (15/247)	6% (1)	5% (3)	7% (4)	6% (4)	7% (3)
Disturbed taste/smell	27% (67/247)	13% (2)	28% (17)	34% (20)	28% (19)	20% (9)
Fatigue	30% (69/231)	- ^a^	21% (13)	31% (18)	33% (22)	36% (16)
Concentration problems	19% (44/231)	- ^a^	13% (8)	19% (11)	21% (14)	24% (11)
Memory problems	18% (42/231)	- ^a^	11% (7)	16% (9)	22% (15)	24% (11)
Sleep problems	5% (13/247)	0% (0)	5% (3)	7% (4)	4% (3)	7% (3)
Headache	11% (28/247)	0% (0)	11% (7)	14% (8)	9% (6)	16% (7)
Dizziness	10% (24/247)	0% (0)	7% (4)	10% (6)	10% (7)	16% (7)
Tingling in fingers	4% (9/247)	0% (0)	0% (0)	2% (1)	4% (3)	11% (5)

*Statistically significant difference at level *P* < 0.05 in univariable analysis using binomial logistic regression with age group 46–60 as reference group.

^a^Children younger than 16 years were not assessed for these symptoms; therefore, *N* = 231 for these categories.

The youngest age group (0–15 years) rarely suffered persistent symptoms (13%, 2/16), whereas 52% (32/61) of young adults aged 16–30 years who were home-isolated for mild to moderate initial illness had persistent symptoms, the most common being disturbed taste and/or smell (28%), fatigue (21%), dyspnea (13%) and impaired concentration (13%) and memory (11%) (Table 2). In these young adults, comorbidity was not significantly associated with persistent symptoms (33% versus 31%, *P* = 1) or fatigue (47% versus 27%, *P* = 0.2), although numbers of subjects were low.

**Fig. 2:**
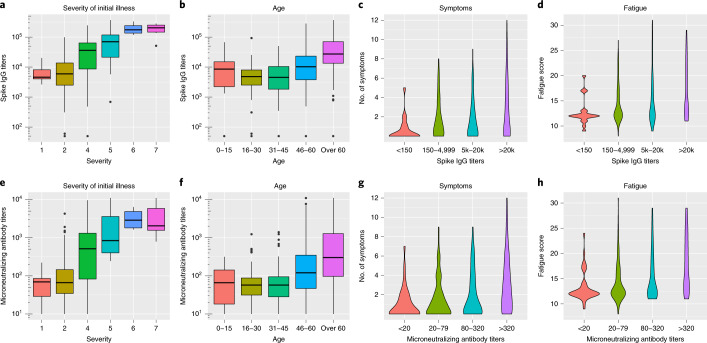
Six-month follow-up of patients with COVID-19 in Bergen, Norway. The relationship of severity of initial COVID-19 illness and of age with anti-SARS-CoV-2 spike (**a** and **b**) and microneutralizing antibody titers (**e** and **f**) at 2 months. The relationship of antibody titers at 2 months with number of persistent symptoms (**c** and **g**) and total fatigue score according to the Chalder scale (**d** and **h**) at 6-month follow-up. The 13 symptoms used are listed in Table 2. The severity of illness was as follows: 1—asymptomatic (*n* = 5); 2—home-isolated with symptoms (*n* = 242); 3—hospitalized without medical needs (*n* = 0); 4—hospitalized with medical needs (*n* = 31); 5—hospitalized needing O_2_ (*n* = 24); 6—hospitalized needing non-invasive ventilation (*n* = 4); and 7—hospitalized needing respirator (*n* = 6). The cohort was divided into 15-year age groups: 0–15 (*n* = 16), 16–30 (*n* = 65), 31–45 (*n* = 69), 46–60 (*n* = 90) and >60 (*n* = 72). **a**, **b**, **e** and **f** show median spike IgG and microneutralizing antibody titers (horizontal line), 25% and 75% quantiles (box), plus 95% confidence intervals (lines) with outliers (dots). **c** and **d** are violin plots of number of symptoms (up to 13) or fatigue score (values 0–33) divided into four categories of spike IgG: <150 (*n* = 21), 150–4999 (*n* = 96), 5,000–20,000 (*n* = 103) and >20,000 (*n* = 92). 5k, 5,000; 20k, 20,000. **g** and **h** are violin plots of four categories of microneutralizing titers: <20 (neg *n* = 42), 20–79 (*n* = 107), 80–320 (*n* = 92) and >320 (*n* = 71). O_2_, supplemental oxygen.

**Extended Data Fig. 1:**
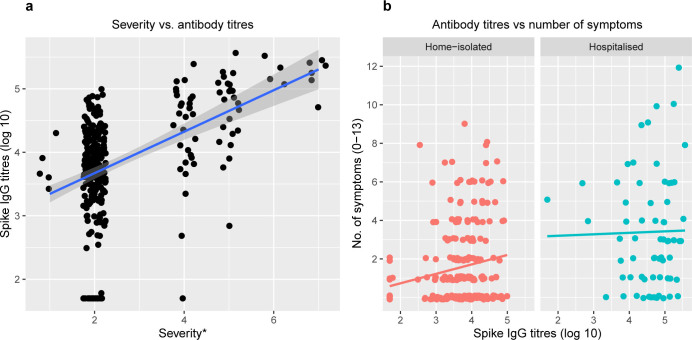
Correlations between severity of acute illness, antibody titres at 2 months and number of symptoms at 6 months follow-up. Panel a is a scatter plot showing the correlation between severity of initial illness and the Spike IgG antibody titres (log 10) at 2 months follow-up. Panel b shows the correlation between Spike IgG antibody titres (log 10) at 2 months follow-up and the number of symptoms at 6 months follow-up, stratified by severity of initial illness, that is home-isolated versus hospitalised patients. A regression line calculated by linear model is shown in each graph with standard error represented by the shaded area.

Convalescent antibodies reach a plateau approximately 1–2 months after infection, providing a general marker for the magnitude of the immune response^16^. SARS-CoV-2 spike protein specific IgG and microneutralizing antibody titers detected after 2 months were significantly higher in hospitalized patients than home-isolated patients (*P* < 0.001; Extended Data Fig. 1). Increased antibody titers at 2 months were associated with the severity of initial illness, older age and higher BMI in multivariable analysis (Table 3 and Fig. 2; severity score adapted from Beigel et al.^17^).

**Table 3:** Factors associated with increasing convalescent antibody titers in COVID-19

		Geometric mean ratio (CI) *P*	
	*n* (%)	Unadjusted	Adjusted
Total	312 (100%)		
Female sex	160 (51%)	0.63 (0.41–0.97) **0.036**	0.81 (0.56–1.18) 0.276
Older age (by 10-year intervals)		1.50 (1.35–1.67) **<0.001**	1.23 (1.09–1.38) **<0.001**
BMI		1.17 (1.12–1.23) **<0.001**	1.05 (1.00–1.11) **0.035**
Comorbidity			
Asthma/COPD	38 (12%)	1.70 (0.88–3.28) 0.111	
Hypertension	35 (11%)	5.43 (2.82–10.46) **<0.001**	1.74 (0.89–3.43) 0.108
Chronic heart disease	22 (7%)	4.68 (2.06–10.64) **<0.001**	0.94 (0.41–2.16) 0.891
Rheumatic disease	20 (6%)	1.67 (0.70–4.01) 0.249	
Diabetes	13 (4%)	3.57 (1.23–10.37) **0.020**	1.47 (0.59–3.71) 0.409
Immunosuppression	11 (4%)	1.51 (0.47–4.83) 0.488	
Current or prior smoker	96 (31%)	1.57 (0.99–2.50) 0.055	
Severity of initial illness		2.12 (1.82–2.48) **<0.001**	1.67 (1.34–2.07) **<0.001**
Days in hospital		1.10 (1.07–1.13) **<0.001**	1.01 (0.97–1.04) 0.679

Associated factors were analyzed by linear regression with log-transformed antibody titers as response variables and reported as geometric mean ratios with 95% confidence intervals (CIs) and *P* values. Factors with significance level *P* < 0.1 in univariable analysis were included in the multivariable analyses. For factors with significance level *P* < 0.05, the *P* values are shown in bold.

Increased antibody titers as well as pre-existing lung disease were independently associated with both persistent fatigue and total number of symptoms at 6 months in multivariable analysis (Table 4 and Fig. 2c,d,g,h). Severity of initial illness was associated with persistent fatigue and weakly associated with total number of symptoms (Table 4).

**Table 4:** COVID-19 patient factors associated with increasing number of symptoms and higher fatigue score at 6-month follow-up—negative binomial regression analysis

		Number of symptoms (0–13)^a^			Fatigue score (0–33)^b^	
	*n* (%)	RR (CI) *P*^c^	aRR (CI) *P*^d^	*n* (%)^b^	RR (CI) *P*^c^	aRR (CI) *P*^d^
	*N* = 312			*N* = 293		
Female sex	160 (51%)	1.28 (0.95–1.73) 0.101	1.35 (1.01–1.81) **0.040**	149 (51%)	1.09 (1.01–1.16) **0.018**	1.09 (1.02–1.16) **0.014**
Older age (by 10-year intervals)		1.18 (1.06–1.28) **<0.001**	1.08 (0.98–1.19) 0.092		1.03 (1.01–1.05) **0.002**	1.00 (0.98–1.02) 0.924
BMI		1.04 (1.01–1.08) **0.016**	1.00 (0.97–1.04) 0.876		1.01 (1.00–1.02) **0.048**	1.00 (0.99–1.01) 0.715
Comorbidity						
Asthma/COPD	38 (12%)	2.00 (1.33–3.07) **0.001**	1.57 (1.05–2.37) **0.031**	37 (13%)	1.22 (1.11–1.34) **<0.001**	1.14 (1.03–1.25) **0.008**
Hypertension	35 (11%)	1.44 (0.93–2.30) 0.114		34 (12%)	1.13 (1.02–1.26) **0.017**	1.01 (0.90–1.13) 0.902
Chronic heart disease	22 (7%)	1.70 (1.01–3.02) 0.057	1.23 (0.71–2.18) 0.460	21 (7%)	1.20 (1.06–1.35) **0.005**	1.08 (0.94–1.23) 0.295
Rheumatic disease	20 (6%)	1.35 (0.77–2.50) 0.321		20 (7%)	1.15 (1.01–1.30) **0.038**	1.05 (0.92–1.18) 0.460
Diabetes	13 (4%)	1.33 (0.67–2.87) 0.438		13 (4%)	1.14 (0.97–1.34) 0.098	1.06 (0.91–1.23) 0.445
Immunosuppression	11 (4%)	1.18 (0.56–2.76) 0.679		10 (3%)	1.12 (0.93–1.34) 0.232	
Current or prior smoker	96 (31%)	1.18 (0.86–1.63) 0.305		95 (32%)	1.05 (0.97–1.12) 0.230	
Severity of initial illness		1.28 (1.14–1.44) **<0.001**	1.17 (1.00–1.37) 0.062		1.08 (1.05–1.10) **<0.001**	1.06 (1.02–1.10) **0.004**
Days in hospital		1.02 (1.00–1.05) 1.016	0.99 (0.97–1.02) 0.525		1.01 (1.00–1.01) **0.002**	1.00 (0.99–1.00) 0.464
Spike IgG titer at 2 months^e^		1.51 (1.26–1.81) **<0.001**	1.25 (1.01–1.56) **0.037**		1.11 (1.07–1.16) **<0.001**	1.07 (1.02–1.12) **0.009**
Microneutralizing antibody titer at 2 months^e^		1.52 (1.25–1.86) **<0.001**	-^f^		1.13 (1.08–1.19) **<0.001**	-^f^

Analysis of associated factors was done by negative binomial regression.

aRR, adjusted rate ratio; RR, rate ratio.

Statistical significance at the level of *P* < 0.05 is shown in bold text.

^a^Patients were assessed for 13 symptoms mentioned in Table 2.

^b^Chalder fatigue score is validated only for patients aged ≥16 years (*n* = 293); possible fatigue scores range from 0 (no fatigue) to 33 (worst possible fatigue).

^c^Neurological illness (*n* = 8) and malignancy (*n* = 5) were not significantly associated with the outcomes and were not included in the table.

^d^Factors with statistical significance of <0.1 were included in the multivariable analysis.

^e^SARS-CoV-2 spike protein antibody titers, log_10_ transformed.

^f^Microneutralizing antibody titers were omitted owing to collinearity with spike IgG antibody titers.

Post-viral fatigue has been reported afer SARS infection^7^ and other viral infections^11–13^. To assess fatigue, we used the Chalder fatigue score, which is validated for adults^18,19^. Fatigue is defined as a total bimodal score of 4 or higher on 11 questions. Thirty percent (69/231) of home-isolated patients, 16 years of age or older, had fatigue at 6 months compared to 63% (39/62) of hospitalized patients. Severe fatigue at 6 months, defined as bimodal score ≥4 + total ordinal score ≥23, was present in 7% (17/231) of home-isolated and 24% (15/62) of hospitalized patients (Supplementary Table 1). In home-isolated patients, the most frequent symptoms of physical fatigue (questions 1–7) were tiredness (35%, 81/231), increased need for rest (30%) and lack of energy (29%); the most common symptoms of mental fatigue (questions 8–11) were difficulties finding words (23%), difficulties concentrating (19%) and memory problems (18%) (Supplementary Table 1).

In patients 16 years of age or older, fever during acute illness, severity of initial illness and female gender were associated with fatigue at 6 months in binomial logistic regression (Supplementary Table 2). In addition to female gender, pre-existing lung disease, severity of acute illness and increased convalescent antibody titers were independently associated with increasing fatigue score at 6 months in multivariable analysis (Table 4 and Fig. 2d,h).

Despite the correlation between severity of initial disease and antibody titers at 2 months, both factors were independently associated with features of long COVID in multivariable analysis (Table 4 and Supplementary Table 2). In stratified analysis of 242 home-isolated patients with low to moderate symptoms, increased antibody titers remained associated with number of symptoms (odds ratio (OR) = 1.56, confidence interval (CI) 1.23–1.96) and fatigue score (OR = 1.07, CI 1.02–1.12), although the association was not statistically significant for fatigue as a dichotomous variable (OR = 1.48, CI 0.98–2.31).

## Discussion

Our study is novel in assessing long COVID symptoms, not only in hospitalized patients but also in young patients and home-isolated patients with milder disease. A strength is a near-complete, geographically defined cohort of both antibody- and reverse-transcriptase polymerase chain reaction (RT-PCR)-positive patients from the first pandemic wave, including all severities of disease, in an immunologically naive population. The small subgroups are a limitation of this study, and our findings should be confirmed in larger cohorts.

We found that a large proportion of survivors of COVID-19 in our cohort had persistent symptoms 6 months after their initial illness. Although it has previously been reported that patients hospitalized for severe COVID-19 frequently suffer long-term symptoms^20–23^, we found that more than half of home-isolated, mildly to moderately ill patients with COVID-19 still suffered symptoms 6 months after infection. It is worrying that non-hospitalized, young people (16–30 years old) suffer potentially severe symptoms, such as concentration and memory problems, dyspnea and fatigue, half a year after infection. Particularly for students, such symptoms might interfere with their learning and study progress.

The high prevalence of persistent fatigue in patients with COVID-19 is striking and appears higher than observed after common infections, such as influenza, Epstein–Barr virus mononucleosis and dengue^11–13^. Data from Norway have previously shown slightly lower chronic fatigue prevalence (11%) in the general population^24^ than in the present household controls (14%), who were younger and had fewer comorbidities than infected patients but were sampled at the same time. However, this apparent difference might be a coincidence owing to low numbers. Our finding that women had higher prevalence of fatigue concurs with results from an earlier study in the general Norwegian population^24^. The association between severity of illness and persistent symptoms agrees with data from hospitalized patients with COVID-19 (ref. ^20^). As the respiratory tract is the main target organ for COVID-19, our finding of an association between underlying chronic lung disease (mostly asthma) and persistent symptoms, including fatigue, is not surprising.

The association between severe initial disease and increased antibody titers at 2 months could be due to higher viral load, which could trigger the immune system more profoundly^25^. The finding of increased convalescent antibody titers with increasing age could be explained by more severe disease in older people, as age is a known strong risk factor for severe COVID-19. However, by contrast with the immunosenescence observed after influenza infection in the elderly, multivariable analysis indicated that age and severity of illness were independently associated with increased antibody titers. These findings call for enhanced surveillance of COVID-19 mass vaccination programs. Home-isolated patients aged 16–30 years with mild COVID-19 are at risk of long-lasting dyspnea and cognitive symptoms. Considering the millions of young people infected during the ongoing pandemic, our findings are a strong impetus for comprehensive infection control and population-wide mass vaccination.

## Methods

### Ethical considerations

All participants, or their guardians for children younger than 16 years old, provided written informed consent. The study was approved by the Regional Ethics Committee of Western Norway (no. 118664). No compensation was provided to participants beyond reimbursing travel costs to the clinic.

### Study population

As part of a prospective cohort study with long-term follow-up, we consecutively enrolled home-isolated patients diagnosed with COVID-19 in the period 28 February to 4 April 2020, during the first wave of the pandemic in Bergen, Norway. For hospitalized patients, we accepted longer inclusion due to delay in hospitalization, up to 6 May. The study population included SARS-CoV-2 RT–PCR-positive patients diagnosed at Bergen Municipality Emergency Clinic and those admitted to the two neighboring city hospitals: Haukeland University Hospital and Haraldsplass Deaconess Hospital. Household contacts of identified patients were invited to participate in the study as secondary cases (seroconverters) or seronegative controls^15^. All registered patients with SARS-CoV-2 were invited to participate. The initial rate of participation at inclusion was 92% (278/302); at 2 months, the rate of participation was 88% (336/381, numerator and denominator includes seropositive household members at this time point); and at 6 months, the rate of participation was 82% (312/381). Convalescent serum samples were collected 2 months after infection for detection of anti-SARS-CoV-2 antibody titers.

All testing in Bergen was centralized to the Emergency Clinic and the city’s two hospitals, allowing recruitment of all consenting patients diagnosed with COVID-19 in Bergen. In total, 3,319 patients were tested by RT–PCR in the Municipality Emergency Clinic during the period. Of these, 228 tested positive by RT-PCR, and six were excluded because their address was outside of Bergen or because they were admitted to hospital and were categorized as hospitalized. All suspected cases seen at the Municipality Emergency Clinic were examined clinically and diagnosed by medical staff. Patients with moderate disease were sent home for home isolation, or, if considered severe, they were hospitalized. Most of the hospitalized patients were diagnosed upon admission. Nasopharyngeal swabs were collected for laboratory confirmation. Patients were telephoned with their results of the RT–PCR test, which was conducted at the reference microbiology laboratory in the tertiary hospital. All confirmed home-isolated patients were contacted by telephone with an invitation to join the study. An additional 79 patients were identified through investigation of seroconversion of household members of RT–PCR-positive patients. Telephone interviews were conducted by medical staff to collect clinical and demographic data. Participants attended the University Clinic at 2 months (6–8 weeks) and 6 months (±1 month) for follow-up appointments with medical staff where they were interviewed about long-term symptoms.

### Clinical data

All consenting patients attended a follow-up clinic and were interviewed by medical staff at baseline, 2 months and 6 months. They provided demographic information; clinical information on symptoms at baseline and 6-month follow-up; and information on potential risk factors, including comorbidities and use of medication. Specific symptoms recorded during acute illness included fever, cough, dyspnea, fatigue, myalgia and headache. Participants were asked if they had any comorbidities and, if so, which specific comorbidities from the following: asthma, chronic obstructive lung disease, chronic heart disease, hypertension, chronic liver disease, kidney disease, neuromuscular disease, dementia, rheumatic disease, active cancer, other severe chronic disease and immunosuppressive conditions, including genetic immunodeficiency, HIV, organ transplant, and cytostatic or other immunosuppressive treatment. Data were collected on severity of initial illness—that is, need for hospitalization, symptoms during acute illness and need for non-invasive ventilatory support or respirator treatment^17^. Radiological investigations were conducted only on hospitalized patients. At 6-month follow-up, all participants aged 16 years or older were invited to complete a validated fatigue questionnaire containing 11 key questions according to the Chalder fatigue scale^18,19^. Fatigue was defined as a total dichotomized score of 4 or higher. Severe fatigue was defined as fatigue plus a total Chalder score of 23 or higher.

### Laboratory methods

Diagnosis of COVID-19 was based on RT–PCR on samples from nasopharyngeal swabs and on serological evidence of SARS-CoV-2 antibody positivity^26^. Serum samples were collected 2 months after infection for detection of anti-SARS-CoV-2 antibody titers and stored at −80 °C until analyzed. Samples were heat-inactivated for 1 h at 56 °C before analysis in duplicate by a two-step ELISA for detecting SARS-CoV-2-specific IgG antibodies (Southern Biotech, cat. no. 2040-05) to the receptor-binding domain (RBD) (screening 1:100 dilution) and the spike protein (confirmation from 1:100 in five-fold dilutions). Endpoint titers were calculated as the reciprocal of the serum dilution giving an optical density value of 3 standard deviations above the mean of historical pre-pandemic serum samples (*n* = 128)^26^. Sera with antibodies against the RBD were tested in a microneutralization assay using the local isolate hCoV-19/Norway/Bergen-01/2020 (GISAID accession ID EPI_ISL_541970) in a certified Biosafety Level 3 laboratory as previously described^27^. Briefly, sera were tested in duplicate in doubling dilutions starting from 1:20 dilution and mixed with 100 TCID_50_ viruses, followed by incubation with rabbit monoclonal IgG against SARS-CoV2 NP (Sino Biological, cat. no. 40143-R019-100) and biotinylated goat anti-rabbit IgG (H+L) (Southern Biotech, cat. no. 4050-08) and extravidin–peroxidase (Sigma-Aldrich, cat. no. E2886). The microneutralizing antibody titer is the reciprocal of the serum dilution giving 50% inhibition of virus infectivity. For all control individuals, negative serology was confirmed at 6 months.

### Statistical analysis

Data were entered using electronic case report forms in REDCap (Research Electronic Data Capture, Vanderbilt University). All analyses were conducted in R version 4.0.3 (www.r-project.org), and graphs were produced in R using the ggplot and gridExtra packages. Patients who responded to the questionnaire were included in the analysis, and results are presented as percentages with means or medians and 95% CIs. In univariable analysis, categorical variables were compared using the chi-square test and binomial logistic regression and presented with ORs, 95% CIs and *P* values.

Multivariable analysis was performed by binary logistic regression for dichotomous outcome variables (Supplementary Tables 1 and 2). We used negative binomial regression employing the MASS package in R to analyze factors associated with numeric outcome variables (Table 4)—that is, ‘number of symptoms’, encoded as integers from 0 to 13, according to symptoms listed in Table 2, and fatigue score according to the Chalder scale encompassing values from 0 to 33. For convalescent antibody titer as outcome variable, we log-transformed the titer values to obtain near-normal distribution and performed linear regression and reported results as unadjusted and adjusted geometric mean ratios for univariable and multivariable analysis, respectively (Table 3). In Tables 3 and 4, we included a priori potential risk factors of interest but omitted rare occurrences, and, for multivariable analysis, we included gender as well as variables that had a significance level of *P* < 0.1 in univariable analysis. Owing to strong collinearity between spike IgG and microneutralizing antibody titers, and because microneutralizing antibodies are a proportion of total IgG, we omitted microneutralizing antibodies from the multivariable analysis.

The analysis focuses on the potential effect of antibody titers as an exposure variable on fatigue and symptom score, respectively, as outcome variables. We assessed the confounding and effect size modification of all other exposure factors that were significant in univariable analysis.

Severity of illness was classified using an eight-category ordinal scale, as previously published^17^. The categories are as follows: 1—not hospitalized and no limitations of activities; 2—not hospitalized, with limitation of activities, home oxygen requirement or both; 3—hospitalized, not requiring supplemental oxygen and no longer requiring ongoing medical care (used if hospitalization was extended for infection control or other non-medical reasons); 4—hospitalized, not requiring supplemental oxygen but requiring ongoing medical care (related to COVID-19 or to other medical conditions); 5—hospitalized, requiring any supplemental oxygen; 6—hospitalized, requiring non-invasive ventilation or use of high-flow oxygen devices; 7—hospitalized, receiving invasive mechanical ventilation or extracorporeal membrane oxygenation; and 8—death.

### Reporting Summary

Further information on research design is available in the Nature Research Reporting Summary linked to this article.

## Online content

Any methods, additional references, Nature Research reporting summaries, source data, extended data, supplementary information, acknowledgements, peer review information; details of author contributions and competing interests; and statements of data and code availability are available at 10.1038/s41591-021-01433-3.

## Supplementary information

Supplementary Information Supplementary Tables 1 and 2.

Reporting Summary

Supplementary Tables Supplementary Tables 1 and 2 in Excel format

## Data Availability

Small subgroups of patients make the risk of identification of sensitive data of individual patients possible; therefore, the data are not openly accessible.

## Code availability

The R code used to generate all results in this paper is publicly available on GitHub (https://github.com/BC19RG/NatureMedicine2021).

## References

[1] Gupta A (2020). Extrapulmonary manifestations of COVID-19. Nat. Med..

[2] Meeting the challenge of long COVID. *Nat. Med.* **26**, 1803 (2020). 10.1038/s41591-020-01177-6 33288947

[3] Zhou F (2020). Clinical course and risk factors for mortality of adult inpatients with COVID-19 in Wuhan, China: a retrospective cohort study. Lancet.

[4] Menni C (2020). Real-time tracking of self-reported symptoms to predict potential COVID-19. Nat. Med..

[5] von Weyhern CH, Kaufmann I, Neff F, Kremer M (2020). Early evidence of pronounced brain involvement in fatal COVID-19 outcomes. Lancet.

[6] Venkatesan P (2021). NICE guideline on long COVID. Lancet Respir. Med..

[7] Lam MH (2009). Mental morbidities and chronic fatigue in severe acute respiratory syndrome survivors: long-term follow-up. Arch. Intern. Med..

[8] Kerr WR, Coghlan JD, Payne DJ, Robertson L (1966). The laboratory diagnosis of chronic brucellosis. Lancet.

[9] Ayres JG, Smith EG, Flint N (1996). Protracted fatigue and debility after acute Q fever. Lancet.

[10] Hanevik K (2014). Irritable bowel syndrome and chronic fatigue 6 years after giardia infection: a controlled prospective cohort study. Clin. Infect. Dis..

[11] White PD (2001). Predictions and associations of fatigue syndromes and mood disorders that occur after infectious mononucleosis. Lancet.

[12] Buchwald DS, Rea TD, Katon WJ, Russo JE, Ashley RL (2000). Acute infectious mononucleosis: characteristics of patients who report failure to recover. Am. J. Med..

[13] Seet RC, Quek AM, Lim EC (2007). Post-infectious fatigue syndrome in dengue infection. J. Clin. Virol..

[14] Geense, W. W. et al. New physical, mental, and cognitive problems 1-year post-ICU: a prospective multicenter study. *Am. J. Respir. Crit. Care Med.*10.1164/rccm.202009-3381OC (2021). 10.1164/rccm.202009-3381OC 33526001

[15] Kuwelker, K. et al. Attack rates amongst household members of outpatients with confirmed COVID-19 in Bergen, Norway: a case-ascertained study. *Lancet Reg. Health Eur.* **3**, 100014 (2021). 10.1016/j.lanepe.2020.100014 PMC8009692 33871470

[16] Robbiani DF (2020). Convergent antibody responses to SARS-CoV-2 in convalescent individuals. Nature.

[17] Beigel JH (2020). Remdesivir for the treatment of Covid-19—final report. N. Engl. J. Med..

[18] Chalder T (1993). Development of a fatigue scale. J. Psychosom. Res..

[19] Wessely S, Powell R (1989). Fatigue syndromes: a comparison of chronic ‘postviral’ fatigue with neuromuscular and affective disorders. J. Neurol. Neurosurg. Psychiatry.

[20] Huang C (2021). 6-month consequences of COVID-19 in patients discharged from hospital: a cohort study. Lancet.

[21] Bellan M (2021). Respiratory and psychophysical sequelae among patients with COVID-19 four months after hospital discharge. JAMA Netw. Open.

[22] Sykes DL (2021). Post-COVID-19 symptom burden: what is long-COVID and how should we manage it?. Lung.

[23] Venturelli S (2021). Surviving COVID-19 in Bergamo province: a post-acute outpatient re-evaluation. Epidemiol. Infect..

[24] Loge JH, Ekeberg O, Kaasa S (1998). Fatigue in the general Norwegian population: normative data and associations. J. Psychosom. Res..

[25] Fajnzylber J (2020). SARS-CoV-2 viral load is associated with increased disease severity and mortality. Nat. Commun..

[26] Amanat F (2020). A serological assay to detect SARS-CoV-2 seroconversion in humans. Nat. Med..

[27] Trieu MC (2021). SARS-CoV-2-specific neutralizing antibody responses in Norwegian health care workers after the first wave of COVID-19 pandemic: a prospective cohort study. J. Infect. Dis..

