# Supplementary material for: Long COVID in a prospective cohort of home-isolated patients
Source: Nat Med. 2021 Jun 23;27(9):1607–13. doi: 10.1038/s41591-021-01433-3 (PMC8440190; doi:10.1038/s41591-021-01433-3)
Supplement: Supplementary file 1 — Supplementary Tables 1 and 2. [file 41591_2021_1433_MOESM1_ESM.pdf]

nature portfolio

https://doi.org/10.1038/s41591-021-01433-3

Supplementary information

Long COVID in a prospective cohort of home-isolated patients

In the format provided by the authors and unedited

Long Covid in a prospective cohort of home-isolated individuals

Bjørn Blomberg, Kristin Greve-Isdahl Mohn, Karl Albert Brokstad, Fan Zhou, Dagrun Waag Linchausen, Bent-Are Hansen, Sarah Lartey Jalloh, Therese Bredholt Onyango, Kanika Kuwelker, Marianne Sævik, Hauke Bartsch, Camilla Tøndel, Bård Reiakvam Kittang, Bergen COVID-19 Research Group, Rebecca Jane Cox, Nina Langeland

Supplementary material

Page 2 Supplementary Table 1 | Fatigue score at six months follow-up in COVID-19 patients (n=293) and seronegative controls (n=43) older than 15 years

Page 3 Supplementary Table 2 | Factors associated with fatigue in 293 COVID-19 patients at six months follow-up

Supplementary Table 1 | Fatigue score at six months follow-up in COVID-19 patients (n=293) and seronegative controls (n=43) older than 15 years

Characteristic	Seronegative exposed controls % (n)	All COVID-19 patients % (n)	Hospitalised COVID-19 patients % (n)	Home-isolated COVID-19 patients % (n)	Hospitalised versus home-isolated OR (CI)	aOR (CI)
	N=43	N=293	N=62	N=231		
Female	65% (28)	51% (150)	45% (28)	53% (122)	0.74 (0.42-1.29)	0.50 (0.24-1.00)*
Age, median (IQR)	42 (26-53)[a]	47 (33-60)	55.5 (45-68)	45 (30-55)[b]	1.24 (1.20-1.29)*	1.04 (1.02-1.06)*
Fatigue	14% (6)[a]	37% (108)	63% (39)	30% (69)[b]	3.98 (2.23-7.25)*	
Severe fatigue	2% (1)	11% (33)	24% (15)	7% (17)[b]	4.02 (1.86-8.64)*	
Total score, mean (CI)	12.8 (11.9-13.7)[a]	15.4 (14.8-15.9)	18.6 (17.3-20.0)	14.5 (13.9-15.0)[b]	1.29 (1.20-1.38)*	
Physical fatigue, mean (CI)	1.1 (1.0-1.2)	1.3 (1.3-1.4)	1.7 (1.5-1.8)	1.3 (1.2-1.3)[b]	1.36 (1.06-1.67)*	
Tiredness	19% (8)[a]	42% (122)	66% (41)	35% (81)[b]	3.62 (2.02-6.63)*	0.64 (0.16-2.31)
Need for more rest	19% (8)[a]	39% (114)	71% (44)	30% (70)[b]	5.62 (3.08-10.63)*	5.89 (1.69-22.14)*
Feeling sleepy	16% (7)[a]	32% (94)	53% (33)	26% (61)[b]	3.17 (1.78-5.69)*	0.86 (0.32-2.30)
Problems starting things	12% (5)[a]	26% (77)	48% (30)	20% (47)[b]	3.67 (2.03-6.66)*	0.85 (0.28-2.56)
Lack of energy	19% (8)[a]	35% (104)	58% (36)	29% (68)[b]	3.32 (1.87-5.97)*	0.84 (0.24-2.77)
Reduced muscle strength	5% (2)[a]	24% (70)	53% (33)	16% (37)[b]	5.97 (3.26-11.07)*	2.13 (0.80-5.73)
Feeling weak	5% (2)[a]	22% (63)	45% (28)	15% (35)[b]	4.61 (2.49-8.58)*	1.01 (0.34-2.92)
Mental fatigue, mean (CI)	1.0 (1.0-1.1)	1.3 (1.2-1.3)	1.5(1.4-1.6)	1.2 (1.1-1.2)	1.26 (0.99-1.59)	
Difficulties concentrating	5% (2)[a]	26% (77)	53% (33)	19% (44)[b]	4.84 (2.67-8.85)*	3.36 (1.22-9.51)*
Slips of the tongue	5% (2)	11% (31)	23% (14)	7% (17)[b]	3.67 (1.68-7.96)*	1.73 (0.59-5.05)
Difficulty finding words	5% (2)[a]	28% (81)	45% (28)	23% (53)[b]	2.77 (1.53-4.98)*	0.45 (0.16-1.17)
Memory problems	2% (1)[a]	24% (69)	44% (27)	18% (42)[b]	3.47 (1.90-6.36)*	1.09 (0.38-2.98)

OR odds ratio, aOR adjusted odds ratio from binomial logistic regression, CI 95% confidence interval, IQR interquartile range
* statistically significant difference at level p<0.05.
[a] Significantly lower (p<0.05) in negative controls than in COVID-19 patients.
[b] Significantly lower (p<0.05) in home-isolated than in hospitalised COVID-19 patients.

Supplementary Table 2 | Factors associated with fatigue in 293 COVID-19 patients at six months follow-up

Characteristic	All n	Fatigue % (n)	No fatigue % (n)	OR (CI)	aOR(CI)
	293	n=108	n=185		
Baseline features					
Female sex	150	43% (64)	57% (86)	1.67 (1.04-2.72)*	2.02 (1.13-3.70)*
- Male	143	31% (44)	69% (99)	1	
Age, median (IQR)	293	51.5 (37-61)	45 (30-57)	1.02 (1.00-1.03)*	1.01 (0.99-1.03)
Age categories					
- 16-30 years	65	26% (17)	74% (48)	1	
- 31-45 years	68	35% (24))	65% (44)	1.54 0.74-3.28)	
- 46-60 years	88	40% (35)	60% (53)	1.86 (0.94-3.81)	
- Over 60 years	72	44% (32)	56% (40)	2.26 (1.11-4.73)*	
BMI, median (IQR)	293	25.3 (23.1-27.7)	24.7 (23.0-27.2)	1.03 (0.97-1.09)	0.99 (0.91-1.07)
Any comorbidity	133	47% (62)	53% (71)	2.16 (1.34-3.51)*	
- None	160	29% (46)	71% (114)	1	
Asthma, COPD[a]	37	62% (23)	38% (14)	3.31 1.62-6.75)*	2.17 (0.97-4.96)
- No lung disease	256	33% (85)	67% (171)	1	
CHD	21	62% (13)	38% (8)	3.03 1.21-7.56)*	2.23 (0.72-7.25)
- No CHD	272	35% (95)	65% (177)	1	
Hypertension	34	50% (17)	50% (17)	1.85 (0.90-3.79)	
- No hypertension	259	35% (91)	65% (168)	1	
Diabetes	13	46% (6)	54% (7)	1.49 (0.40-5.39)	
- No diabetes	280	36% (102)	64% (178)	1	
CKD	1	100% (1)	0% (0)	NA	
- No CKD	292	37% (107)	63% (185)	1	
Immunosuppression	10	60% (6)	40% (4)	2.64 (0.61-13.09)	
- None	283	36% (102)	64% (181)	1	
Smoker[b]	95	41% (39)	59% (56)	1.31 0.79-2.17)	
- Never smoked	196	35% (68)	65% (128)	1	
- NA	2	50% (1)	50% (1)		
Initial illness					
Hospitalised	62	63% (39)	37% (23)	3.98 (2.21 – 7.16)*	
- Home-isolated	231	30% (69)	70% (162)	1	
Days in hospital, mean (CI)	293	2.9 (1.6-4.3)	1.6 (0.5-2.7)	1.02 (0.99-1.06)	0.97 (0.92-1.02)
Symptoms					
Fever	56	54% (30)	46% (26)	2.39 (1.31-4.36)*	2.68 (1.24-5.90)*
- *No fever*	209	33% (68)	67% (141)	1	
Dyspnoea	147	43% (63)	57% (84)	1.76 (1.05-2.94)*	0.86 (0.44-1.66)
- *None*	117	30% (35)	70% (82)	1	
Cough	191	38% (72)	62% (119)	1.12 (0.64-1.96)	
- *None*	74	35% (26)	65% (48)		
Fatigue	239	37% (89)	63% (150)	1.12 (0.48-2.62)	
- *None*	26	35% (9)	65% (17)	1	
Myalgia	155	40% (62)	60% (93)	1.41 (0.84-2.36)	
- *None*	109	32% (35)	68% (74)	1	
Headache	167	40% (66)	60% (101)	1.29 (0.76-218)	
- *None*	95	34% (32)	66% (63)	1	
Severity of illness					
- Asymptomatic	3	67% (2)	33% (1)	4.81 (0.45-104.45)	
- Symptomatic home-isolated	228	29% (67)	71% (161)	1	
- Hospitalised	28	61% (17)	39% (11)	3.71 (1.67-8.58)*	
- Need for oxygen	24	71% (17)	29% (7)	5.84 (2.40-15.69)*	
- Need for NIV	4	75% (3)	25% (1)	7.21 (0.90-147.17)	
- Need for respirator	6	33% (2)	67% (4)	1.20 (0.16-6.31)	
Severity, median (IQR)[c]	293	2 (2-4)	2 (2-2)	1.49 (1.23-1.83)*	1.99 (1.37-2.96)*
Spike antibodies[d]	293	4.1 (3.9-4.2)	3.7 (3.6-3.9)	1.71 (1.25-2.39)*	1.23 (0.81-1.92)
Microneutralising antibodies[d]	293	2.2 (2.1-2.4)	1.9 (1.8-2.0)	1.78 (1.27-2.52)*	
Acute complications					
ARF	8	38% (3)	62% (5)	1.03 (0.24-4.39)	
- None	285	37% (105)	63% (180)	1	
Hypotension	3	100% (3)	0% (0)	NA	
- None	290	36% (105)	64% (185)	1	
Lung failure	23	52% (12)	48% (11)	1.98 (0.84-4.65)	
- None	270	36% (96)	64% (174)	1	
Antibiotic use	31	55% (17)	45% (14)	2.28 (1.08-4.84)*	0.42 (0.10-1.75)
- None	62	35% (91)	65% (171)	1	

OR: odds ratio, aOR: adjusted odds ratio from multivariable analysis by binomial logistic regression, CI: 95% confidence interval, BMI: body mass index, COPD: chronic obstructive lung disease, CHD: chronic heart disease, CKD: chronic kidney disease, NIV: non-invasive respiratory support, ARF: acute renal failure. Variables were assessed by Poisson regression.

*Statistical significance at the level $p<0.05$

[a] Twenty of 23 had asthma

[b] Current or prior smoker.

[c] Severity on an eight-category ordinal scale.

[d] Means and 95% confidence interval of log10-transformed convalescent antibody titres 2 months after initial illness.
